# The Outlook of Healthcare Providers on the Involvement of Diabetic Patients as Health Promoters for Diabetes Prevention Among Their Family Members: A Qualitative Study

**DOI:** 10.7759/cureus.42108

**Published:** 2023-07-19

**Authors:** Ghaida Alsulami, Muna Alharbi, Mona Alanazi, Abeer Aseeri, Eman Bajamal

**Affiliations:** 1 Faculty of Nursing, Umm Al-Qura University, Mecca, SAU; 2 College of Nursing, King Saud bin Abdulaziz University for Health Sciences, Riyadh, SAU; 3 College of Nursing, King Khalid University, Abha, SAU; 4 College of Nursing, King Saud bin Abdulaziz University for Health Sciences, Jeddah, SAU

**Keywords:** health care provider, patient with diabetes, diabetes prevention, qualitative study, health promotion

## Abstract

Background and objective

Examples of patients becoming health promoters for diabetes prevention in their own families, although few, are on the rise. Nevertheless, despite this increase in patient involvement in diabetes prevention, there is scarce research regarding healthcare providers’ perspectives on the active engagement of patients as health promoters for their family members. In light of this, we aimed to explore the perspectives of healthcare providers working at primary health clinics regarding patient involvement in diabetes prevention among their own family members and close relatives.

Methodology

This study was conducted between July and December 2022 at the Primary Healthcare Clinics at King Abdulaziz Medical City, Ministry of National Guard Health Affairs, in Riyadh and Jeddah, Saudi Arabia. Semistructured interviews based on purposive sampling were conducted with 13 participants, and their data were thematically analyzed.

Results

Two main themes emerged from the interviews: the patients' readiness to be health promoters and the willingness of healthcare staff to support promoter patients. Healthcare providers perceived the involvement of diabetes patients in promoting the health of their family members and close relatives as beneficial; however, several barriers may prevent these patients from becoming effective health promoters.

Conclusions

Healthcare providers understand the significance of involving patients with diabetes as health promoters for their family members and close relatives. Patients can offer unique insights into the lived experience of diabetes management, as well as provide practical advice for lifestyle adjustments. Nevertheless, healthcare providers should also recognize the limits of patients’ knowledge and skills and ensure that patients receive proper training and support to serve as effective health educators.

## Introduction

Diabetes is a major health issue that affects millions of people worldwide. The number of individuals affected by diabetes globally is expected to increase from 463 million in 2019 to 592 million by 2035 [1]. An analysis by the International Diabetes Federation (IDF) revealed that the Middle East and North African (MENA) region had the world’s highest diabetes prevalence (12.2%), and this is expected to rise to 13.3% by 2030. The high prevalence of diabetes in this area is linked to the region’s rapid economic expansion, a change to a more sedentary lifestyle among the population, urbanization, and the westernization of dietary habits with a shift to fast food [2]. In 2014, Saudi Arabia accounted for 17-18% of the total number of diabetics in the MENA region, placing it almost fifth in rank among the 20 countries in the MENA region [3]. A cross-sectional study in Saudi Arabia conducted from 2007 to 2009 on the prevalence of diabetes involving 18,034 individuals older than 30 years identified a prevalence rate of 25.4% and a significantly higher prevalence in urban than in rural areas [4].

Diabetes has a known and significant impact on the well-being and quality of life of humans, and the impacts of diabetes can include other serious illnesses. For example, diabetes is the leading cause of acute kidney failure, cardiovascular events, serious infections, hyperosmolar coma, and diabetic ketoacidosis [5], all of which may lead to ICU admissions [5]. The rate of ICU admissions has increased for patients with diabetes [5], and this could also significantly increase healthcare costs, in part due to the need for higher staffing ratios. Overall, diabetes imposes social and economic burdens around the world that seriously impact society as well as healthcare systems [6]. The cost is significantly increased for patients who require longer durations of intensive care treatment, as this increases the burden on hospital finances [6].

More than 95% of people with diabetes have type 2 diabetes (T2DM) [7]. This type of diabetes arises largely as a result of unhealthy nutritional habits, excess body weight, and physical inactivity. Healthcare providers working in primary healthcare clinics, therefore, can play an important role in addressing, controlling, and preventing diabetes among their clinical patients. They are in a position to educate patients about programs for the prevention and control of T2DM, such as screening, educational programs, lifestyle modifications, nutritional programs, pharmacologic intervention, and prevention of cardiovascular diseases [8]. The paradigm of diabetes management has shifted in recent years to focus on empowering the patient with diabetes to manage the disease successfully and to improve their own quality of life [9]. There has been a growing interest in involving patients with diabetes as promoters of good health among their own relatives by sharing information about the health risks of T2DM and the lifestyle changes necessary to prevent this disease.

In recent years, the role of patients, especially those with chronic diseases, in disease management has moved beyond their own care and has now extended to them assuming positions as potential deliverers of care or, at a minimum, as contributors to the improvement of care delivery and research initiatives [10]. Most people suffering from a chronic disease can take responsibility for their own disease management with the guidance of health professionals, and this improves their condition and quality of life [10]. However, in addition to their primary role in the management of their own disease, some patients have been steered toward undertaking a role in the education of the wider public, including their own close friends and family, to better inform those around them of the disease’s risks. Chronic diseases, such as asthma and diabetes, often require the patient to acquire important knowledge and skills for the successful management of their disease and to understand the nature of and treatment for their health issues. However, for the most part, they have to be willing to use and update this knowledge appropriately [11].

Active patient involvement in professional health education has been extensively recommended in international research and studies. This involvement could take the form of patients teaching medical students about patient-centered care and inter-professionalism, as well as encouraging a greater focus on the community, competence in cultural practices, and ethics [12]. Empowering diabetic patients to discuss the risks of the disease with family members has been suggested as a powerful tool for the prevention of diabetes. Patients are well-positioned to influence the lives of their family members and close friends in a way that makes them effective in raising awareness of diabetes and subsequently encouraging the adoption of healthy lifestyle behaviors. Relatives of patients with diabetes are considered at an increased risk of developing type 2 diabetes themselves [13]; therefore, healthcare providers within primary healthcare settings can play an important role in encouraging patients with diabetes to be health promoters who can inform their relatives of the health risks and lifestyle changes that are necessary to prevent the development of diabetes. 

Some reports have shown that patients engaged in this type of involvement can serve as health promoters regarding diabetes prevention in their own families [14]. However, despite this growing encouragement of patient involvement in diabetes prevention, there is a lack of information regarding the perspectives of healthcare providers toward this idea of active engagement of patients as health promoters for their family members. To the best of our knowledge, no contemporary study has yet explored the views and attitudes of healthcare providers at primary healthcare clinics regarding soliciting the involvement of patients with diabetes as health promoters for diabetes prevention in their own families. A deep understanding of healthcare providers’ views and attitudes is valuable since these are the people who engage with patients with diabetes in healthcare settings on a regular basis and thus play a considerable role in diabetes prevention. Therefore, the aim of this study was to conduct a qualitative exploration of healthcare providers’ views and perceptions about involving patients with diabetes as health promoters for diabetes prevention among their own family members and close relatives, as well as to explore the barriers to and facilitators of participation by patients with diabetes as health promoters focused on diabetes prevention.

## Materials and methods

Study design

This qualitative descriptive study involved in-depth, semi-structured interviews to gain insight into the perceptions of healthcare providers working at primary healthcare clinics regarding patient involvement in diabetes prevention among their family members and close relatives.

Sample selection and context

This study was conducted at the Healthcare Primary Healthcare Clinics at King Abdulaziz Medical City, Ministry of National Guard Health Affairs, in Riyadh and Jeddah, Saudi Arabia. Primary healthcare clinics are staffed by professional healthcare teams that work to meet people’s health needs, ranging from health promotion to disease prevention, treatment, rehabilitation, and palliative care.

The participants were selected using purposive sampling that included a mix of different healthcare professional disciplines, varying levels of experience, and assorted positions/roles within the healthcare primary clinics. The aim was to obtain a sample of healthcare providers that would represent a broad spectrum of perspectives regarding the involvement of patients with diabetes as health promoters of diabetes prevention within their own families. The study inclusion criteria were as follows: healthcare providers who were formally registered, involved in the care of patients with diabetes in a primary healthcare clinic, and willing to participate in this study. Potential participants were sent invitations and contacted face-to-face or via email by the research teams to ask if they would be interested in taking part in the study. The healthcare providers who expressed an interest were given an information sheet and offered an appointment to see the research nurse to discuss their involvement, check their eligibility, and consent to the study. The interviews continued until data saturation was reached, and were then stopped when no new themes emerged from them. In total, 13 individuals were interviewed, including one physician, nine registered nurses, and three registered diabetes nurse educators.

Data collection

Individual, semi-structured interviews were conducted using a predesigned framework to collect data. The interview framework for the semi-structured interviews used in this study incorporated a set of semi-structured questions that sought to promote efficiency and consistency within the available timeframes for both the interviewer and the interviewees (See Appendix).

The interview framework was developed based on the existing literature and experts’ opinions and was particularly influenced by the Health Belief Theory (HBT) [15]. To fulfill the research objectives, the interview questions were framed in accordance with key HBT components: susceptibility, severity, benefits, barriers, cues to action, and self-efficacy. Perceived severity comprised an individual’s attitude and perceived risk of acquiring diabetes because of an existing patient with diabetes in the family. At the beginning of the interviews, participants were asked general questions regarding their views of the risks to family members due to the presence of a patient with diabetes. Specific questions were then asked to unearth their beliefs regarding the likelihood that the patient’s family, offspring, and other relatives were at risk of developing diabetes; this encompassed the second component in the HBT [i.e., the belief of consequence (perceived susceptibility)]. Specific questions regarding the respondents’ perception of the benefits of involving patients with diabetes as health promoters of diabetes prevention for their own family members and close relatives were also included to involve the third component in the HBT [potential positive benefits of action (perceived benefits)]. Later in the interviews, participants were asked specific questions about barriers to and facilitators of the involvement of patients with diabetes in diabetes prevention initiatives for their family members and close relatives. This was an attempt to understand the respondents’ perspectives, which pertain to the fourth and fifth components of the HBT [perceived barriers to action and exposure to factors that prompt action (cues to action)]. The interview guide ended with questions about the respondents’ willingness to encourage their patients with diabetes to be health promoters for their family members and close relatives, and this comprised the last component of HBT (self-efficacy).

To advertise the interviews, a recruitment flyer was displayed on the noticeboards of the departments within the healthcare promotion clinic and distributed to the healthcare providers via an email sent to a clinic’s administration. The email also contained an explanatory statement and the consent form. To reach healthcare providers who failed to check their email inboxes regularly, healthcare providers who did use their work email addresses regularly were asked to share a digital copy of the recruitment flyer with their peers on social media (e.g., WhatsApp). The flyer contained brief information about the study as well as the researcher’s email and contact number to enable potential participants to seek more information about the study. Healthcare providers who showed interest and wanted to know more about the study were encouraged to read the explanatory statement, which fully described the research procedures and the efforts to protect their anonymity. Any further questions were answered and clarified prior to the healthcare providers agreeing to participate. The interviewers then arranged a date and time for the ensuing interviews with the voluntary participants. The healthcare providers were required to sign the consent form prior to attending the interview. The interviews were carried out by two researchers, who were extensively trained to conduct qualitative interviews.

Data were collected through individual semi-structured interviews (from July to December 2022) conducted by two co-researchers at the participants’ workplaces. Each interview lasted 30-45 minutes on average. Each interview was digitally recorded and transcribed verbatim into English by a professional transcriber.

Data analysis

Data were analyzed manually based on the six phases of thematic analysis described by Braun and Clarke [16]. The transcribed interviews were read several times by each author to obtain an understanding of all the data. The first author coded the data, which was rechecked and discussed by another author to ensure consensus on the coding practices. The first author also generated initial themes from the data and then consulted with the second author for feedback and consensus on the naming and grouping of sub-themes. The authors then compared and discussed the sub-themes to reach an agreement.

Research credibility was ensured by taking several steps, such as prolonged engagement and rechecking the audio recordings of the participants several times. The authors aimed for trustworthiness in accordance with standard criteria for qualitative research. The thorough description of the design and method, as well as the description of the setting and participants and the choice of quotations to strengthen the results, enriched the credibility of the results. The authors engaged in discussions on the material throughout the progression of the analysis, which also enhanced its credibility.

To analyze the obtained data, the researcher first repeatedly listened to the interviews several times before transcribing them. The initial encoding was then performed, and codes with similar meanings were listed under a sub-theme with an appropriate label. The sub-themes with similar meanings were then classified as a theme. The initial codes, sub-themes, and themes were then reviewed and relabeled. Finally, the report was written.

To ensure the trustworthiness of the data, the researchers were granted sufficient time to collect and go back and forth between the data to ensure that the material was acceptable. The sub- and main themes were formed by moving the initial codes between the sub-themes several times. Dependability was achieved through the development of an audit trail and by having a qualitative expert review the findings.

Ethical considerations 

The institutional review board (IRB) approval was obtained from King Abdullah International Medical Research Center (KAIMRC). All participants were informed of the objective of the study and their right to withdraw from the study at any time during data collection. Informed consent was obtained from those who agreed to participate in person. All participants were reassured that their responses would be kept confidential and their identities would not be disclosed in any resulting publications. The study presented no risk or discomfort other than the foreseeable inconvenience regarding the time taken by the participants to complete the interviews. Pseudonyms were used during the transcription process in place of the participants' real names.

## Results

In this study, 13 participants were interviewed and the resulting data were analyzed. All the participants were female, and their ages ranged between 27 and 56 years. Most of the participants were registered nurses, and a few were diabetes educators. The analysis process focused on understanding the healthcare providers’ perspectives regarding involving patients with diabetes as health promoters for diabetes prevention among their own family members and close relatives. Two main themes emerged from the interviews: the patient’s readiness to be a health promoter and the willingness of healthcare staff to support promoter patients (Table 1).

**Table 1:** Patients with diabetes as health promoters for their family and close relatives: healthcare providers’ perspective

Theme	Sub-theme
Patient's readiness to be a health promoter	Benefits of being a health promoter
	Barriers and facilitators to being a health promoter
The willingness of healthcare workers to support promoter patients	Healthcare provider's confidence
	Barriers to engaging patients
	Requirements for a successful initiative

1. Patients’ readiness to be a health promoter

The first theme that emerged was the healthcare providers’ perspectives on patients’ readiness to be health promoters for their family members and close relatives. Their perspectives were based on their work experiences with patients with diabetes. Most of the participants were interested in the discussion on this topic since they understood the risks posed by diabetes disease on the families of patients with diabetes. They perceived diabetes as a critical health problem and understood that family members of patients with diabetes are also susceptible to diabetes.

They also believed that health promotion could decrease family members’ susceptibility to diabetes. The majority of the participants discussed factors that may affect patients’ readiness to undertake an action, such as promoting the health of their family members and close relatives. These factors are outlined in two dimensions: (1) benefits and (2) barriers and facilitators that the patients may perceive or encounter if they become diabetes healthcare promoters.

1.1. Benefits of Being a Health Promoter

Many of the participants considered that the inclusion of patients with diabetes to promote the health of their family and close relatives would be beneficial. Most of the participants agreed that the experiences and journeys of patients with diabetes in the process of their disease management would help them become health promoters. Because patients with diabetes experience many challenges and understand how it feels to live as patients with this disease, they would be able to translate that into health promotion. Moreover, patients with diabetes have a greater awareness of the disease than those who do not live with diabetes. One of the participants explained:

“I think it is very important that they will be health promoters to their relatives and their family members because at least they have the experience of being a patient with diabetes. They know about the experiences of treatment and management that people with diabetes mellitus may have in and out of the hospital. This experience itself makes it easy for patients to communicate it to their relatives.” (Participant 1).

Another participant expressed the following view:

“He (patient) can be a health promoter. He can express himself; if he explains his lifestyle, the medication, and what he has to go through in the process of being a patient with diabetes, it helps others. He can be like a blogger or something like that . . . I think it’s a good idea and could also help the patient. Being a health promoter will also help society because you can see nowadays that some bloggers can change a person’s life.” (Participant 3).

One participant shared her views on the benefits of health promotion by patients with diabetes among their family members and close relatives and she felt that it could lead to reducing the number of critical cases that were admitted to hospitals because of diabetes complications and added that it would also help the patient gain family support:

“It saves money . . . I think the first benefit is that it will decrease the number of visits to the clinic. I think also that the admissions to the hospital will be low . . . The first thing I think is that the family will really know what their father or mother is going through, so maybe they will support the person who has diabetes more; they will be more caring toward them.” (Participant 9).

Some of the participants highlighted the love of family that motivates someone to take care of their family members and close relatives. This love would be the reason that patients with diabetes would seek to increase their families’ awareness and protect their kids from being harmed by diabetes. One participant clarified this by saying:

“I think the first thing is the love for their family. This will actually help facilitate; because if you love your family, you will want to let them know about the risk of having this problem or condition. Because of that, they will be able to really share their experiences and hope that they can prevent their relatives from having this kind of disease.” (Participant 2).

One participant suggested that an additional benefit of including the patient as a diabetes health promoter was that it would enhance the patient’s adherence to their own diabetes management. The health promoter role requires someone who is capable of being a role model and cares about how people see them.

“I think it turns to the point that the patient takes care of their family members. They would not want their family members to experience what they have experienced. Aside from that, the patients can use themselves as an example to family members to indicate that they should take care of themselves and their health.” (Participant 6).

1.2. Barriers and Facilitators to Being a Health Promoter

From the perspective of healthcare providers, several barriers existed that made them doubt patients’ readiness to be health promoters for their family members and close relatives. These barriers should be eliminated and the patient’s health promotion activities should be facilitated. One of the most important barriers is lack of knowledge; most of the participants agreed that patients lack knowledge and have insufficient information about the management of diabetes symptoms and complications, which would be a barrier to them being health promoters. More importantly, if the patients did not adhere to or commit to their medication or the management of their diet, they would not be able to encourage others to take safe measures to prevent diabetes. In this case, the patient would not be a good role model for their family and close relatives.

“If the patient with diabetes is non-compliant with his own treatment . . . another thing is that the patient may lack knowledge or have incomplete knowledge about diabetes itself. If that is the case, it would not be appropriate for him to be a health promoter.” (Participant 4).

Some of the participants highlighted the age of the patient as a potential barrier to being a health promoter. They stated that most of the patients who visited a primary healthcare center for diabetes were elderly, suggesting that those patients had insufficient knowledge and did not usually have the capacity to learn or teach. These older patients normally came with their relatives to help them understand how they could adhere to treatment and manage the diseases that afflict them. In this way, they may indirectly engage their family members and close relatives in health promotion about diabetes.

“I think it’s challenging since it depends on the patient’s background, education, and willingness. It depends on where you work. I work at the primary healthcare clinic, which is like a clinic, and we have a lot of patients who are coming . . . we are dealing with old patients, basically they are uneducated . . . so, from my experience, family members are not engaged in their healthcare.” (Participant 7).

One of the participants stated that, depending on the patients' characteristics, there may be barriers to them becoming health promoters:

“I think not all . . . not all patients with diabetes can be health promoters. Just my own opinion, especially if a patient has limited knowledge or a poor educational background, meaning that there are things that they cannot explain further if they’re asked some questions regarding the condition. Another factor that I can think of is the age of the patient, i.e., if the patient is too young or too old to explain and provide health education to the family members.” (Participant 5).

One of the barriers that patients may encounter is that their family members and close relatives may not be sufficiently willing to listen to the patient’s advice. The patients also may not receive adequate support from healthcare providers to equip them with enough information to educate their family members and close relatives. A good level of understanding of diabetes disease prevention and treatment would help patients become health promoters.

“I think it is about the level of patient understanding-whether the patient really understands the causes and effects of diabetes and the capacity of the patient to convey the information to promote wellness with regard to the disease information. The capacity to convey information to family members may be limited by . . . maybe they live far from their family members or the patient does not wish to share what they are experiencing. So, these are factors that I think will discourage the patient from sharing the information with a family member.” (Participant 6).

2. Willingness of healthcare workers to support promoter patients

The second theme that emerged from the interviews was the willingness of staff to support their patients to serve as diabetes health promoters for their families and close relatives. This theme can be divided into three sub-themes: the healthcare provider’s confidence, barriers to engaging patients, and the requirements for a successful initiative.

2.1. Healthcare Providers’ Confidence

Most of the participants felt confident that they would encourage their patients with diabetes to be involved in communicating health promotion experiences to their family members and close relatives. Some of them stated that they were 100% sure that they would motivate their patients to promote the healthcare of their families and close relatives.

“We can say that we are confident enough because we really want to share with all the people, especially those who are at risk of developing diabetes. So, it is really a good program to train all patients with diabetes to be promoters for their family members and relatives.” (Participant 2).

“For me, I’m willing because I have taken a diabetes education course and I have worked in this area for one year. However, it depends on the patient and the relative, and whether they want to listen to me and start. If they do, this will truly encourage me; otherwise, I will just give them the important information or follow up later.” (Participant 8).

However, even though a few of the participants perceived that diabetes was a threat to the family members of patients with diabetes, they did not show interest in the patient health promoter program and considered that it would not work, or they were more interested in promoting other things to foster community health. These participants thought that this program would not help large segments of the community and that the focus of the programs would be on particular families rather than the community as a whole.

“I am not that interested . . . to tell you the truth. I tried once, then I ditched the idea, they are-you know-old people like my old mother, my old father. They do not accept everything, and this here is one thing that they do not accept . . . The occupation of the patient matters, also the population matters, all these things affect it. If you want to do something realistic and there is a benefit, you have to talk about all the aspects.” (Participant 7).

In contrast, most of the participants were willing to support the initiative of making patients with diabetes to be health promoters, but they advised that it needs to be supported. As one participant stated:

“I’m willing. It’s a good idea. It can help us, and it can help to improve a good lifestyle for the patients, their families, and the community . . . Maybe easy communication between the healthcare providers and the patients and the availability of resources. It’s really a good idea and something good that you are thinking about or studying because we need it. We need family members and patients to help us improve and prevent people from getting this disease. So, it is a good idea and we will think about initiating it in our healthcare organization.” (Participant 12).

2.2. Barriers to Engaging Patients

Occasionally, barriers are encountered not only by patients but also by healthcare providers, which would impact their willingness to support patients’ role in improving the health of their family members and close relatives. Most healthcare providers in Saudi Arabia are non-Arab; therefore, the language barrier is the most critical barrier that could inhibit staff from providing their patients with enough knowledge for health promotion. In this case, the healthcare provider’s role is confined to caregiving and does not extend to health education and promotion.

“Actually, we do not give that much education because we are just giving care and do not provide much education to the patient. They come to the clinic and just receive information about the diet and the food. They are not given advice about how to take care like that because this is what the diabetes educators do.” (Participant 13).

One of the participants expressed that she could not translate important information for patients and therefore could not help them prepare to provide health advice to their family or close relatives:

“For me, talking about myself, I think the number one issue is the language barrier because I do not speak Arabic that well. I can manage, but not when explaining things to people in Arabic. So, I think one is the language barrier and also insufficient knowledge about diabetes itself.” (Participant 4).

Another barrier encountered by healthcare providers besides language is lack of time. Primary healthcare centers where the data was collected are busy clinics that serve a large number of patients daily, and hence the staff could not find the time to work on equipping their patients with information on diabetes to be health promoters.

“Well, I think because we are here in Saudi Arabia, the language barrier is very significant. It is hard for us to explain our point of view, the medical terms that we should like to convey to our patients are complicated to explain in terms that patients understand, especially in the Arabic language or an Arabic dialect. Aside from that, there are time constraints; it is hard to have the time to explain to the patient or family member. For example, we would have to go into the community, to their house setting, because, in the hospital, we have limited time to explain to them.” (Participant 6).

Although most of the participants knew that diabetes was risky for patients’ families and understood that susceptibility to the disease is higher in families with a history of diabetes, they perceived the non-support of superior staff in the center as a barrier. This is a barrier because no work can be done with patients in the absence of sufficient time, funds, resources, and appreciation.

“She (the nurse) should be supported to deliver this program because if she felt supported and appreciated by her superior, she would work on it. You know, once upon a time, I ran a diabetes program for patients with diabetes. But if I am not supported on this by my superior, I will not work on it . . . If I am not appreciated when I’m putting effort then I will not, I will not be encouraged to do it . . . If there is no support, if there is no appreciation and we are not encouraged, then why should we do it? It’s just putting extra hard work on us.” (Participant 7).

*2.3.* *Requirements for a Successful Initiative*

This initiative of supporting patients to be health promoters for their family members requires many things if it is to be successful. Most of the participants were confident that they would support such an initiative because they believed in the importance of protecting families of patients with diabetes from this disease. The participants put forward some strategies to help patients become health promoters.

“One hundred percent sure because it’s an important thing for the patients to educate themselves and spread the knowledge . . . I think the fastest way would be to give patients some brochures that describe simple topics or main topics in a way that is straightforward and easy to understand.” (Participant 10).

The participants agreed that patients need a lot of information to be equipped with the knowledge and resources to prepare them for this health promoter role. They agreed that they would be willing to support patients and train them on how to be successful healthcare promoters.

“I think one of the factors that could help is that the patient must be knowledgeable enough about their condition because health education is difficult . . . So I think being knowledgeable about the condition, the medications they are receiving, and how to take care of themselves . . . To do this, the patient must have support . . . support from the physician or a nurse within the circle of healthcare.” (Participant 5).

“Healthcare staff need to provide the patient with instructions on how to be a health promoter and help them to understand new things that they are unfamiliar with, at a level that is simple and can be understood by the patient.” (Participant 11).

## Discussion

The aim of this study was to explore the perceptions of healthcare providers working at primary healthcare clinics regarding patient involvement in diabetes prevention among their own family members and close relatives. Patient-centered education has been reported to be an effective approach for empowering patients with diabetes to better manage their condition. The findings of this study mostly supported the idea of patients with diabetes serving as health promoters for their family members, because doing so has several benefits that help the patients themselves in their self-management of diabetes. In this study, the participants agreed that patients with diabetes can play a critical role as health promoters for their family members, including their spouses, children, and other family members, and they believed this would improve patient outcomes. Given that family members of patients with diabetes are particularly at increased risk of developing type 2 diabetes [13], some interventions are needed to prevent diabetes among these individuals. Previous research has focused on the role of family members in caring for and educating their loved ones with diabetes [17,18]. Additionally, the present findings indicate that healthcare providers, including physicians, nurses, and diabetes educators, believe that patients with diabetes can play a significant role in educating their family members and close relatives about the disease. In addition, patients can provide their family members with realistic expectations regarding the disease, explain complex disease mechanisms, and educate them about healthy lifestyle changes.

In this study, healthcare providers also reported that patients can provide insights into the emotional and psychological impacts of living with diabetes that are not always learned from textbooks. This is consistent with a study by Park et al. (2018), who found that involving family members in diabetes education programs improved patients’ psychological well-being and quality of life [19]. Patients' lived experiences and their role in providing social support in healthcare settings by sharing their knowledge, insights, and personal experience can help healthcare providers deliver more effective diabetes education to other patients and their families. It has been suggested that patients can provide context to complex disease mechanisms, thereby helping to bridge knowledge gaps associated with diabetes management.

Overall, healthcare providers recognize the potential of patients with diabetes to be health promoters for their family members and close relatives. They acknowledge that the patients’ unique knowledge and experience can complement traditional diabetes education and thereby personalize care. However, healthcare providers need to ensure that patients receive adequate training and support to be effective health educators and to address the barriers that may limit their education efforts.

Conversely, some healthcare providers in this study expressed concerns about the reliability of diabetes knowledge that is acquired from a patient. They believed that patients who may have lower health literacy may not be effective educators and may transmit incomplete or incorrect information. It should be also noted that family members may have different learning styles and information needs that patients may not be able to provide.

Effective diabetes self-management education often includes educating the patient’s family members and close relatives. This helps to increase the support and engagement of family members in assisting with the patient’s diabetes management. However, barriers that may prevent patients with diabetes from acting as health educators for their family members and close relatives still remain. This discussion also focuses on the barriers that patients with diabetes face when acting as health educators for their family members and close relatives. These barriers include the patient’s knowledge and understanding of diabetes, cultural and social norms, and language and communication.

The primary barrier that patients with diabetes may face is a lack of knowledge and understanding of diabetes. Patients may not fully understand their own disease or may not have received proper education regarding self-management practices. This can limit their ability to adequately educate their family members and close relatives. This finding is similar to a previous study involving patients with diabetes who have low health literacy. It was found that patients with limited health literacy have misconceptions about diabetes that hinder their self-management efforts [20]. These misconceptions create challenges for patients who wish to educate their family members and close relatives effectively.

The second barrier is related to cultural and social norms. Patients may feel uncomfortable discussing their health status with family members or may be hesitant to challenge traditional customs and beliefs that may impact their self-management practices. These cultural and social norms can limit their willingness to share their knowledge and experiences. In terms of cultural and social norms, a study on cultural factors influencing diabetes self-management conducted among Arab immigrants found that cultural factors affected patients' and their families' education about effective diabetes self-management [21].

The third barrier is related to language and communication. Patients with limited proficiency in English or other languages may have difficulty communicating the complexities of diabetes management to their family members and close relatives. Additionally, when language and cultural barriers exist, patients may have a hard time finding available resources for effective communication. The role of language and communication in diabetes education has been explored by previous researchers, through systematic reviews and meta-analyses; they found that patients with limited English proficiency face difficulty in communicating with their healthcare providers and receiving diabetes education [22]. These barriers may also prevent patients from adequately educating their family members and close relatives.

Overall, barriers to effective diabetes self-management education for family members and close relatives are multi-factorial and require comprehensive solutions. Healthcare providers need to consider linguistic, cultural, and social norms that may impact education efforts and work with patients to understand their knowledge and understanding of diabetes. This may help patients address and overcome some of these barriers to diabetes education.

The findings of this study add to the knowledge about the strategies that can help patients with diabetes act as health promoters for their families and close relatives. Patient educators can provide emotional support, social support, and practical advice that can contribute to positive health behaviors and improved diabetes management. Patient educators can also provide practical advice and support for patients concerning nutrition, exercise, blood glucose monitoring, medication management, and insulin administration. This can help patients better manage their diabetes and improve their quality of life. Moreover, patient educators can also provide emotional support and reinforce the importance of personal responsibility and self-care in diabetes management.

This study had some limitations that should be acknowledged. Firstly, the data were collected from a single setting, and hence the results may not be generalizable for other settings. Second, the scope of this study was confined to healthcare providers, and no patients were included. Extending the breadth of inquiry could help us better understand the procedures and problems associated with patient and family engagement. Lastly, we could not include any male participants only one physician participated in this study. Even though the data were homogeneous because of the similar characteristics shared by participants, and this gives strength to the study, the inclusion of males and more healthcare providers may have provided different perspectives on the topic.

## Conclusions

Based on our findings, patient-centered diabetes promotion can provide many benefits for patients with diabetes. Patient educators can provide practical advice and emotional support to other patients, thereby contributing to better diabetes management and improved quality of life. Further research is required to explore the most effective methods for training patient educators and optimizing the delivery of patient-centered diabetes education programs. Healthcare providers recognize the importance of involving patients with diabetes in the role of health promoters for their family members and close relatives. Patients can provide unique insights into the lived experience of managing diabetes and provide practical advice for lifestyle changes. However, healthcare providers should also consider the limitations of patients’ knowledge and skills and ensure that patients receive appropriate training and support to serve as effective health educators. Ultimately, partnerships between healthcare providers, patients, and their family members are essential for achieving optimal outcomes in diabetes management.

Providing effective diabetes self-management education to patients’ family members and close relatives is an important aspect of diabetes care. However, patients with diabetes face several barriers when acting as health educators for their family members and close relatives. To address these barriers, healthcare providers need to provide culturally sensitive diabetes education to patients and their families. They also need to assess each patient’s level of knowledge and understanding of diabetes to ensure that all patients are equipped to provide effective diabetes education to their family members and close relatives. Additionally, healthcare providers need to foster supportive environments in which patients can comfortably and confidently share their experiences and knowledge with their family members and close relatives.

## Appendices

**Table 2:** Interview framework HCP: healthcare provider

Framework	
Participant background information	
Title	
Age	
Sex	
Years of work experience	
Question	Rationale
To what extent can diabetes cause threats to diabetic patients’ family members?	Warm-up questions to examine how the participants perceived threats and harms to diabetic patients' families (perceived severity)
To what extent are the patient’s family members and close relatives at risk for developing diabetes?	To uncover participant’s, beliefs about the likelihood that the patient's family offspring and other relatives will develop diabetes (Belief of consequence (perceived susceptibility)
Based on your views, what are the benefits of diabetic patients being health promoters for their family members and close relatives?	To uncover the HCPs' views of involving diabetic patients as health promoters of diabetic prevention for their family members and close relatives (potential positive benefits of action (perceived benefits)
Based on your views, what are the disadvantages of diabetic patients being health promoters for their family members and close relatives? Can you provide any experience that you hear from your patients that they were health promoters for their family members and close relatives?	These questions are connected to previous questions. It is important to define the disadvantages of a new proposed approach and describe any familiar experience
What factors do you think may obstruct diabetic patients from being health promoters to prevent diabetes among their family members and close relatives?	To identify barriers to the participation of diabetic patients as health promoters of diabetic prevention for their family members and close relatives (perceived barriers to action AND exposure to factors that prompt action (cues to action)
What factors do you think may facilitate/help diabetic patients to be health promoters of diabetes prevention among their family members and close relatives?	To identify facilitators of participation of diabetic patients as health promoters of diabetic prevention for their family members and close relatives (perceived barriers to action AND exposure to factors that prompt action (cues to action)
How willing are you to encourage your patients to be health promoters for their family members and close relatives? Can you explain any barriers or facilitators that may affect your willingness to encourage or involve your patients to be health promoters for their family members and close relatives? How confident do you feel that your division will succeed in involving your patients as health promoters for their family members and close relatives?	To examine participants’ willingness and confidence in their ability in encouraging and involving their patients to be health promoters for their family members (self-efficacy)
